# St. Thomas's Hospital

**Published:** 1831

**Authors:** 


LIII.

ST. THOMAS'S HOSPITAL.

CASES OF FOUL, SLOUGHING, AND CARCINOMATOUS ULCERATION, IN WHICH A SECRET REMEDY, SAID TO BE SPECIFIC, HAS BEEN TRIED.

SOME of our readers have probably heard of the pretensions (we can use no milder term) of Mr. Wall, a person of no education, and unconnected with the medical profession, to cure cancerous and other foul ulcers by a remedy which he has in his possession, and which we are given to understand is a product of the vegetable kingdom in India. For reasons which will appear in the sequel fully satisfactory, we need not however trouble ourselves in enquiring particularly what it is. Through the kindness and liberality of Mr. Green, the merits of the remedy have been tested in this hospital, under the direction of its possessor himself, who had free access to the wards, and who, we believe, was introduced to the notice of Mr. Green by Mr. Callaway and Mr. B. Cooper, of Guy's Hospital. Mr. Callaway has informed us that, in one case of carcinoma, in his private practice, Mr. Wall's dressings at first produced a cleaner and more healthy state of the surface of the ulcer; but this, he observed, was nothing more than many other applications would have done, and how the case has proceeded we have not been able to learn. A case of carcinomatous ulceration of (we think) the mamma under Mr. Cooper, in Guy's, was offered to Mr. W. which he refused to treat, as he considered the disease too far advanced. This happened during last Autumn, prior to the treatment of those we are now about to lay before the public, who will, with ourselves, be convinced by them of the complete inutility of the *nostrum* in question in the hands of its present occupier. We are informed it was Mr. Wall's intention to make known the drug used, if after public trial it should prove efficacious; and therefore, with the laudable motive of endeavouring to discover some curative means for a most painful and irremediable disease, Mr. Green allowed these experiments to be made, which were witnessed by himself and those attending his practice at the hospital. It would be, perhaps, still desirable for the profession to be acquainted with the substance employed, as it might be turned to advantage in the management of certain ulcers. Mr. Wall is utterly ignorant of medicine or surgery, and consequently incapable of judging in what instances the application might be proper.

The subjoined reports will, we trust, be found highly interesting, independently of those reasons just alluded to, which alone would not have entitled them to be related at length.

CASE 1. *Carcinomatous Ulceration of Lower Lip. Failure of Mr. Wall's Dressings and Treatment. Removal of the Symphysis and Anterior Portion of the Lower Jaw.*

William New, æt. 48, admitted into Isaac's Ward, Dec. 9th, 1830.

Nearly the whole of the lower lip is destroyed, to within a short distance of the commissures on either side, by cancerous ulceration. A portion of the lip thus appears taken away in a somewhat semicircular form, the convexity being downwards towards the centre of the chin, there being fully an inch in vertical length from the lowest part of the border of the ulcer to the point we might imagine as the red margin of the lip. The edges of the ulcer are thick, hard, and irregular; in its centre, near to the mucous membrane of the labium attached to the gum, (which membrane is not lost to the same extent as the more superficial parts, having resisted the destructive process longer than the adjacent textures,) is a small slough, but not deep. No discharge takes place from its surface, excepting a thin and colourless exudation, sufficient to prevent its becoming dry. The surrounding parts are remarkably hard and unyielding to the touch, particularly underneath the chin, but no distinct glandular enlargement is perceptible about the neck. Our patient describes the pain he experiences as corresponding with that characteristic of cancer, namely, burning, and lancinating at times; and the intervals of ease have latterly become shorter. He first observed the disease in the lip six months ago, commencing in the form of a scab, which repeatedly fell off, and a fissure then succeeded it; this slowly increased by ulcerative action, accompanied with pain at first but slight; during the last six weeks, however, the progress of the ulceration has been rapid, so that we may say the present sore has been established within that time. The man's constitution seems rather broken up for his years, and his appearance is likewise that of a person beyond his age; but his health, he says, has never suffered, and he now finds no cause for complaint save the local disease.

Dec. 20th. Was visited by Mr. Wall, at the request of Mr. Green; the former expressed his opinion, without any reserve, that he would be able to cure the ulcer. He directed pil. hydr. gr. iv. to be given every night, and magn.

sulph. ℥ss. every third morning. A linseed poultice to the sore.

21st. Mr. Wall applied his dressing this morning. It produced slight pain for the first few minutes, which has now ceased.

23d. Has been daily dressed, with the same effect upon the part as to pain. He has occasionally a darting pain on one side of the lip, but not so severe as previously to the application of the present dressing.

28th. Same dressings have been continued; the surface of the sore has certainly contracted, and has rather the appearance of healthy granulations in some parts. The pill at night is omitted, and he is now taking quinæ sulph. gr. ij. thrice daily, with allowance of ℥ij. of port wine in the twenty-four hours.

31st. The hardness around the ulcer and below the chin is very much diminished, and the sides of the sore are occupied by larger granulations, but in the centre there still remains a superficial slough, which is somewhat bigger at present than a week ago. There is no discharge from the surface, which appears still further contracted. The patient's eyes are red, especially in the morning, and the countenance has a tendency to flushing. Dressed daily by Mr. Wall. Wine and quinia continued.

1831, Jan. 6th. In the centre of the ulcer there is greater loss of substance, and the destroying process has proceeded further towards the gums and mucous membrane of the lip. The surface is also more foul in this situation, and the hardness is equally perceptible as heretofore below the chin. Mr. Wall says his object is to destroy the surface still more, and then cicatrization will rapidly ensue. He applies his vegetable powder, mixed up with lard, and then covers the surface, besmeared with this, with dry lint; the latter again he confines with a strap of plaister, and over the whole a bread poultice is put.

9th. The surface both in the centre and around the ulcer is very much cleaner; and within the last few days there has been scarcely any local pain, although some of a shooting character is occasionally felt in the face, passing upwards to the forehead. Pulvis calaminæ was to-day applied by Mr. Wall.

11th. Powder of arrow-root was last night used locally; the appearance of the ulcer is the same as two days ago.

18th. Ulceration has spread on the upper border of the lip on the right side, but involves merely the mucous covering. It may be said, however, that the diameter of the ulcer is increased nearly half an inch since the patient's admission, not including this superficial spot, which is quite smooth and of healthy appearance. The glands below the ramus of the jaw are perceived to be in some degree swollen.

31st. Latterly there has not been any change effected in the external characters of the sore, but the part has been tolerably free from pain. The hardness of the substance of the lip on both sides of the ulceration is undiminished, and its edges are now rather hard, and similar in aspect to that texture we often see surrounding the callous indolent ulcer of the leg. The same means have been persevered in both locally and generally, and the patient does not complain of any suffering.

Feb. 8th. The part has been dressed three times a day with ung. resinæ, and the former remedy of Mr. Wall. Very little change in the condition of the ulcer is to be noticed, but it seems to have, in some measure, augmented since last report.

15th. Within the last week the ulcer has indeed diminished, but although healed superficially, the hardness remains fully as obvious; the pain is very trifling. Port wine is discontinued.

25th. Mr. Wall has not been able to attend regularly to dress the patient during the last four days, and it would seem that, partly in consequence of this omission, sloughing has occurred in the centre of the ulcer, attended by considerable pain, which extends upwards on both sides of the face. The sore has not spread laterally; it is now dressed punctually.

March 4th. Upon the sound portion of the lip on the left side, a superficial scab has formed, and the ulceration has likewise spread in this direction; its progress is also observable lower down towards the chin. Severe lancinating pain has been present in the whole face as well as in the diseased portion. The patient is feverish, and complains of the want of rest. Mr. Wall continues the same *specific* application, and the poultices of bread.

12th. The cancerous ulceration has been advancing on the left side of the lip, undermining the sound skin; the newly-exposed surface is covered with a dark and characteristic sloughy matter, which possesses the peculiar smell of carcinoma. Aggravation of pain has been co-existent with the diffusion of the disease, and the patient is more distressed and anxious. Mr. Wall has not visited him for nearly a week, and bread poultices are now simply applied.

22d. Some further increase of ulceration is presented to view, but the surface is something cleaner. The pain is nearly constant, though not very severe in degree.

April 5th. Ulcer is now clean, and has not spread since our last account; it is the seat of no pain.

28th. Very little alteration can be discovered, the surface being still free from foulness or sloughs. Poultices alone are used.

May 26th. Great destruction of the lower lip and soft parts inferior to this has recently taken place, under painful and peculiar sloughing ulceration. The ulcer presents an uneven surface of a dark brown colour, and is surrounded by a high and irregular margin. A correspondent increase of general disturbance has been present. Patient is forsaken by Mr. Wall. Poultices are continued.

June 12th. A cleaner surface is now exhibited than at any period since the late extensive loss of substance. The integuments surrounding the ulcer are reddened and thick, and devoid of tenderness. Latterly he has suffered but little. Mr. Green has once proposed to the patient the removal of the diseased textures, with a portion of the lower jaw, (the outer table of which is, in his opinion, implicated, in addition to the periosteum,) and making a new lip from the skin on the side of the neck, but he would not then consent. This was above two months ago, when the ulcerated surface was scarcely half the present magnitude, and in a quiescent state. The same ultimate resource has been a second time submitted to the man's consideration, and it having met entirely with his own concurrence, Mr. Green will in a few days undertake the operation.

17th. Previously to describing the operation, that was practised on this day, we will give a more detailed account of the actual state of disease in the softer parts, and of the appearances indicating the implication of bone. The substance of the inferior labium is totally extinct, and that covering the chin nearly to the lower border of the symphysis is likewise lost, with the exception of an indurated and projecting lump at the left commissure, and about two lines in width. An irregular, cancerous surface is displayed, but tolerably clean, one very minute sloughy point being perceptible in its middle; it possesses very slight fetor, the discharge being scanty; the surrounding integument is condensed in texture, very solid and firm to the touch, and of a peculiarly dark red discolouration. The incisor teeth are loose, blackened, and agglutinated together by morbid tartar. As the anterior and upper portion of the symphysis is covered merely by thin and diseased granulations from the mucous membrane of the alveoli, the periosteum is no doubt affected, if the granulations be not in fact arising from this latter, and consequently the outer table of the inferior maxillary bone is likely to be unsound. The disease is not at this present period in an active state, pain being unimportant, and no loss of substance having recently occurred. Some febrile action was at one time apprehended from flushing of the cheeks, and excited eyes, but this condition has subsided, the latter symptom excepted, which has continued to this date, though it is less remarkable. His rest has been undisturbed, and the appetite, together with the proper performance of the functions of the alimentary canal, is not impaired. There exists no glandular enlargement in the neck, nor has such been noticed but on

Jan. 18th; it disappeared soon afterwards, and was therefore attributable to the local irritation of the diseased lip, and not to malignant absorption.

On consultation with his colleagues, Mr. Green was induced to remove the disease with the requisite portion of the jaw, and then simply to adapt the parts for the present, in order that some adhesions should be formed around and between the extremities of the bone, which would thus acquire a fixed situation, and to which a new lip would be hereafter applicable, in case the cancerous disease should not return. He had ascertained, from careful comparison by means of wax models adjusted to the chin and neighbouring parts, that a very considerable piece of skin would be required for the formation of the lip, that would extend from the os hyoides nearly to the clavicle, if taken from the middle of the neck; therefore it was resolved to procure it from the side in preference to exposing the larynx and trachea anteriorly; and a portion of this size would necessarily demand firm support in its new situation. The idea of completing the whole undertaking at once was for these reasons abandoned, and the steps pursued in the amputation of the maxilla will be now related.

*Operation.* The first incision was made on the right side of the mouth a short distance internally to the angle, (barely half a line,) and carried downwards and forwards, with the convexity looking outwards, to the median line of the neck, about half an inch below the mental symphysis; this was met in the latter situation, where the muscles attached to the bone posteriorly were exposed by the knife, by a corresponding one, commencing at the commissure of the opposite side. Thus, the whole diseased part and the reddened integument around it were of course inclosed in their area. The labial branch of the external maxillary artery was divided on either side, and immediately tied. A third vessel near the centre of the wound, probably given off from the lingual, was also secured on the right side. The bone was in the next place denuded by detaching the enclosed morbid mass in front, and found to be rough in several points; the periosteum was soft and thickened on the left horizontal ramus so far back as the first molar tooth. It was therefore necessary to extract the left second bicuspid; but the membrane was not involved to an equal extent on the right side, from which the first bicuspidatus had been removed previously to the patient's being brought into the operating theatre. The handle of the knife was now passed from below upwards close to the internal surface of the bone into the cavity of the mouth, where it was liberated by a division of the mucous lining, and it was held steadily in this direction during the process of sawing, to protect the tongue. Hey's saw was first applied on the right, and then on the left side, and afterwards a narrow and longer instrument was more efficaciously used. The hard substance being cut through, was gently elevated by the handle of the scalpel, and the entire anterior portion of the lower maxilla between the points above specified, viz. the first left molar and the second right bicuspid tooth, was readily loosened from the horizontal branch of each side, being now retained only by the muscles connected to it. These were cautiously and slowly separated close to the inner side of the mentum by a sharp-pointed and straight bistoury, and in consequence of strong spasm of the muscles of the tongue, which produced coughing and violent expectorating efforts, some delay was necessary, so that all the muscular origins were not disengaged at once. Rather free hæmorrhage took place from the tip of the tongue, which, however, speedily ceased, owing to the very great retraction of the divided muscles. The two remaining portions of the jaw admitted of being approximated within an inch, and about one line from each other, and the interspace was occupied by the tongue, which was now sunk below its natural plane. No other ligature was called for, and the spasmodic action soon terminated. A thick pledget of lint was interposed between the smooth bony ends, in order to keep these last steady, and the parts were further supported externally by three long strips of adhesive plaister, carried from one temple to the other, round the lower part of the face. The external wound presented an interval agreeing with that between the ends of bone, which was thus covered over by plaister and filled with lint. Additional support was also given to the face below by a four-tailed bandage fastened at the vertex. The upper jaw now very considerably overhung the vacuity arising from the removal of so large a portion of the lower, but the dressings afforded comfort to the patient. During the operation, which lasted upwards of half an hour, the poor fellow was extremely quiet, and made scarcely any complaint; even whilst the inferior dental nerves were severed by the saw he did not shriek from the pain. He was occasionally low, but revived by cold water dashed on his face, and a little taken into the stomach.

Being conveyed to bed, he was placed nearly erect as to the upper half of his body, as in this position alone was respiration unaffected. Some slight depression came on about half an hour afterwards, but was of short duration; at this time his breathing was very calm; the pulse, which had previously been full, of considerable power, and at 100, was accelerated and smaller; and a damp and chilly state of skin likewise subsisted during the same period.

*8 o'clock p m.* He breathes freely, and has made no sign of complaint—pulse 100, of moderate strength. Has made water, but in small quantity. No bleeding has taken place. Feels inclined for sleep.

June 18th. Night has been tranquil, and he has slept for some hours; skin rather hot, though moist; pulse 118, full, compressible; face not heated, but somewhat red—urine high-coloured—no stool—dressings easy, and retained in situ. The nourishment taken has consisted of barley-water, given through a common syringe and gum elastic tube, the extremity of which is placed at the back of the tongue, and deglutition appears surprisingly easy.

19th. Has been undisturbed by difficulty of breathing, or uneasiness in the wound. Some little cough has been excited from the accumulation of mucus at the upper part of the pharynx. He slept for many hours last night, and feels refreshed and comfortable. Pulse 92, soft, and moderately full—skin moist, and temperate—no motion has been passed from the bowels. Linseed infusion and milk have been exhibited by the syringe and tube.

20th. No untoward symptom has appeared, he having been troubled with occasional cough only, from the same cause as yesterday. Pulse 88, tranquil, and compressible—skin cool. The whole of the dressings were taken off, the plaisters having become offensive and dirty. The wound was much contracted and perfectly clean at the upper part immediately adjacent to the tongue; lower than this, there is a slough discernible; the external edges of the wound are in a slight degree foul. Lint, broken up into small pieces, was gently inserted into the cavity below the tongue, and the strips of plaister put on in the same manner as before. During the last twenty-four hours he has consumed a pint and a half of milk and linseed tea, and he is now allowed some beef-tea in addition. These fluids are readily swallowed, when thrown into the upper portion of the pharynx. One stool has been obtained this morning from an injection of gruel and common salt.

21st. He has passed three hours in continued sleep during the night; his skin is cool; pulse 88, not so strong. Being supported by pillows, and the spine erect, he breathes naturally. The cough, however, has been more troublesome, and he refers to the inferior part of the trachea as painful, particularly in the act of deglutition. Neither redness, nor heat beyond the ordinary temperature of the surface, is to be detected externally. The adhesive strips are fixed comfortably in their position of yesterday, though moistened by the free escape of saliva from the mouth; they were not interfered with. He has not yet had an alvine evacuation today. Eight leeches were ordered to the throat.

22d. Pain of throat and dysphagia relieved. No unfavourable symptom is to be mentioned. Dressings were again taken away, and similarly re-applied. The slough on the portion mentioned is merely superficial granulations being visible behind it; no suppurative discharge. Bowels acted once last night, and this morning also. Nourishment to be given in the same quantity and manner as heretofore.

23d. Perfectly easy. Pulse 80. Bowels open.

24th. Wound again dressed. The slough alluded to is already nearly cast off; granulations are springing up from the bone on both sides, and in every part of the wound. Little or no suppuration is yet established. The tongue appears in some degree enlarged and rounded towards the apex, and also protruded forwards. He is quite destitute of any constitutional derangement. A fetid and peculiar odour (similar to that from a carcinomatous ulcer) has arisen from the dressings, and is still perceptible. These were put on in the former mode. Soft biscuit, steeped in milk, has been supplied through the tube.

25th. An enema has been administered to open the bowels, and one evacuation solicited. Everything connected with the wound is going on well, but the tongue has become dry at the point and enlarged, from the constant exposure, giving rise to thirst and much local uneasiness. A piece of oiled silk was directed to be placed in front of the mouth and wound, to be secured at the sides of the face to the roller passed over the head, for the purpose of preventing the contact of air. The dressings have already become foul since yesterday; the fetor from the wound is excessive, and, accumulating in the night, it renders the whole apartment greatly contaminated by morning.

26th. The dressings were re-adjusted, and the surface exhibits more conspicuous granulations, which now secrete pus; those from the divided ends of bone are also healthy and abundant. The tongue is not dry to-day. He is able to speak intelligibly, and the efforts are not painful to him. Another purgative injection was this morning required. The straps are applied with pretty firm pressure so as to contract the extent of the wound.

27th. Much the same. Yesterday he had half a pint of porter, which was taken in the same way as the other fluids, and his pulse possesses at present a little more power. He has also beef-tea, with milk and biscuit.

28th. A remarkably florid and healthy appearance is presented by the whole surface of the wound, which is even now considerably diminished; and the patient is much better for the allowance of porter. His speech is daily improving, and the tongue does not project in the same inconvenient manner. Mr. Green gave permission for him to take a little meat, reduced to a pulpy consistence, in his beef-tea. The soft lint is laid upon the granulations, and the edges confined by long pieces of adhesive plaister.

July 1st. An extremely healthy state of wound continues under the same local remedies, and it is further diminished since our last report. The pulse has regained its natural power, and remains at 80. He takes the meat in pulpy form without difficulty, and his bowels are free.

5th. Wound is contracting, and very clean. Patient devoid of uneasiness, or complaint.

12th. Has been doing well in every respect. The surface of the wound is of a florid colour, and becoming less daily. He can now feed himself with a spoon.

14th. Diarrhœa has supervened today, accompanied with a distressing weight and uneasiness in the epigastrium; but there is no abdominal tenderness, and little pain with each evacuation. The condition of the wound is unaffected.

16th. The disturbance of the bowels has ceased under the employment of the common astringent mixture, and he is now free from the concomitant uneasy sensations within the abdomen. During the last week the granulating surface has remarkably lessened, and has cicatrized at the inferior edge, so that nearly the entire wound is internal to the mouth. Dry lint and adhesive plaister are alone used, and daily.

30th. Some little hardened points of granulation have formed at the edges, and have been increasing somewhat in size and number, but the wound itself is reduced almost to nothing. He feeds himself without difficulty, and is permitted to sit up. One black spot is observable at each bony extremity.

August 16th. Two pieces of bone have exfoliated from the ends, which are now covered over. The little projections, like warts, noticed in our last date, have been got rid of by touching them with sulphate of copper, and the cicatrix is perfected. The man is quite recovered, and walks about in the squares of the building. He gradually acquires more power of retaining saliva, and his pronunciation is growing more distinct. The accommodating processes of nature, after wounds and other injuries, are extremely well illustrated in the little deformity at this period manifest. Contraction of the aperture has been continually going on, as well as approximation of the skin from each side to the centre; and a very slight additional portion of integument, if any, on the present chin (may we so call it) will be requisite for the purposes of a lip.

It is with great pleasure we state, that the operation is likely to be followed by the success due to its novelty and boldness, as the cicatrized wound betrays no tendency to returning malignant action. We have been informed of a case of the same disease in which a method nearly similar was adopted many years ago by M. Dupuytren, at the Hôtel Dieu, and with a favourable issue. In those examples of cancer of the lip, where the bone is affected, it is conceived that this operation may advantageously be had recourse to, and therefore we beg to submit the above case to the attention of those surgeons, who are daily called upon to use the knife—the last resource of our art.

Case 2. *Sloughing Ulcer of Alæ Nasi, and foul Ulcer on the Thigh, from Syphilis—Œdematous Swelling of the Lower Extremity. Inutility of the remedies of Mr. Wall.—Cautious Administration of Mercury successful.*

Alexander Braham, æt. 30, was received into Job's ward, under the care of Mr. Green, October 21, 1830.

Situated on the right side of the face, there is a foul and black ulcer, which has destroyed that side of the nose and the adjacent portion of the cheek, and has also formed an aperture through the superior maxillary bone, whereby the external wound communicates with the upper part of the pharynx by the nares. The hard palate is also lost to some extent. Upon the surface of the ulcer there are dark and dry sloughs, which seem but little disposed to separate. There exists, likewise, a circular sore, of most unhealthy appearance, on the left thigh, about its middle. He has general pains of the joints and limbs, and is suffering much from debility; his rest is disturbed at night, when profuse perspirations have frequently taken place; pulse 120, sharp, and irritable, though weak; tongue white, but moist; bowels costive; has been in the habit of taking opium to procure sleep by night. The patient left the hospital about six weeks ago, having staid here in Lazarus ward (venereal) nearly three months; he was then admitted for eruptive disease of the skin, and his mouth was made sore by mercury.

Mr. Green ordered, Opii, gr. ij., o. n. Ext. sarsæ, ʒj. Ex. decoct. sarsæ co. Oss, b. d. Milk diet.

October 29th. There is very little alteration to be observed in the condition of the patient. The ulcer of the nose is in much the same state, as also that on the thigh. Some little rest is procured by the opiate. His strength he finds not improved, and the bowels are costive and irregular. Remedies continued. To have arrow root, and Mist. sennæ comp. occasionally.

Nov. 12th. The nightly perspirations have ceased, but are succeeded by diarrhœa, which is accompanied with considerable griping pains, but no tenderness of the abdomen. The fætor from the nose is somewhat less, but the appearance of the part is not changed; the sore upon the thigh is diminished, and healing in a healthy manner. Pains in limbs are considerably relieved; and having had an extra allowance of meat, he has, previously to the present attack in the bowels, suffered less from debility. Pulse 100, and feeble — tongue clean, a little whitish.

Ordered, Mist. cretæ co. ℥iss. 6tis. horis.

14th. Patient was attacked early in the morning with chilliness and great increase of debility. Swelling and redness, with pain and a severe sense of distention, immediately commenced in the left groin and proceeded rapidly down the limb even to the toes. At 9 o'clock the sister acquainted Mr. Whitfield of the circumstance, and the patient was visited by him at twelve. A powder of rhubarb and calomel was prescribed to be taken instantly, and spirit lotion ordered to be applied cold to the whole limb. We saw him at one o'clock, and there was a very marked change in the man's countenance—it was anxious, and extremely depressed —his manner was hurried and fidgetty —the pulse was 144, very feeble, but regular — tongue dry, and red at the apex. No sickness, nor vomiting had occurred. He had just taken the powder. The left lower extremity was swollen to twice its natural size, from the groin downwards; the surface was of a livid red colour, which was diffused universally over the limb, with several darker and superficial spots on the thigh, not unlike to those of purpura hæmorrhagica, but occupying merely the cuticle and not being circular. There was exquisite tenderness on the slightest touch, yet no alteration in temperature. The sore, which yesterday looked remarkably healthy, was of a dark crimson hue, almost black, having a blueish circumference; and its secretion was suppressed. The limb felt cold to the patient, especially at the toes, but was not painful. No pulsations of the artery at the groin were perceptible; firm pressure was not made with this view, as it would have caused unnecessary agony.

Was ordered, in addition to the above, Mist. pot. citrat. efferv., 4tâ. q. h.

8 *o'clock, p. m.* Limb is in the same state, excepting that the spots mentioned above have increased somewhat in size, and are now visible on the leg and foot. Pulse 144, weaker than in the morning. No motion has yet taken place from the bowels — there is no sickness—urine very scanty, and highly tinged—tongue as in the morning. To have an injection immediately, and continue cold lotion.

15th. 9, *a. m.* Pulse 168, fluttering —countenance, however, less depressed —tongue unchanged. He got no rest during the night. Swelling and redness of the limb rather less, and the spots are not so conspicuous—pressure can now be borne. Bowels have acted twice since the glyster was thrown up, which also brought away a copious stool. No difference in the sore upon the face.

Wine, ℥j. 6tis horis. Quinæ sulph., gr. ij., 4tis. Cont. opii, gr. ij., o. n.

3, *p. m.* Pulse varies very much in number, being at one time beyond 120, and at another not more than 100; it is excessively feeble. The leg is easy, but the surface very cold. Lotion omitted.

16th. Patient easy, having passed a good night; pulse as yesterday—limb of same size. An abscess appears to be forming a short distance above the heel, there being a soft prominence in this situation. Mr. Green visited him, and ordered wine and nourishment, with fomentations and flannel to the whole extremity.

Ammon. subcarb., gr. x. Tinct. opii, ♏v. Mist. camphoræ, ℥iss, 6tâ. q. horâ.

17th. Passed a tolerable night, and very little change has ensued—limb not so frigid, and the swelling noticed yesterday is subsiding. A large piece of bone, nearly black, has exfoliated from the palate through the nose. The ulcer has not increased in dimensions.

18th. Pulse 120, irregular, and very feeble. He has, nevertheless, slept well, and his tongue is less red. More tumefaction is observable above the heel — the limb in other respects is not changed. Bowels act freely, and the stools are watery.

19th. A superficial slough has separated from the sore upon the thigh, and left a healthy granulating surface. The patient feels generally better, but not in any important degree, and takes nutriment in good quantity. Pulse more regular, 110—bowels continue relaxed; the stools are of natural colour, and not preceded by pain. Mist. cretæ, c. ℥iss. Tinct. catechu, ʒj. 6tâ. q. h. Cont. cætera.

Nov. 21st. Pulse 96, regular, and much stronger—tongue not red, nor dry—bowels quiet. Foot and lower part of leg have become considerably enlarged from œdema. The swelling and discoloration superiorly have very much diminished, and there is no tenderness here on pressure. He rests better, and has no nocturnal sweatings. Ulcer on nose is not cleaning, but appears rather larger, and continues horribly offensive.

23d. Tumefaction is still subsiding, and the patient much improved. Ulcer of thigh is of healthy aspect, and ordered to be strapped. He complains of want of rest only, but his appetite has increased. Extr. conii, gr. v., ter die.

24th. Some swelling and tenderness exist at the back part of the opposite thigh, and three or four tuberculated scabs are upon this limb. Pulse 120, rather irregular; tongue clean, and moist.

27th. Foot and ancle are much less; the leg and thigh are returned to their natural size; and the sore is looking healthy, and diminishing.

Dec. 1st. There is no enlargement of the left lower extremity remaining; the sore is healthy, but its granulations are very small. He complains more of the other thigh. The ulceration upon the face has rather spread latterly. He is ordered to fumigate the nose.

4th. He thinks the sloughing ulceration of the nose is checked, with diminution of pain in that part. His health is amended—pulse 100, regular, and more powerful—nightly rest is better.

Mutton chop. Porter, ℔j. daily.

7th. The tuberculated spots on the right thigh are sloughing, but not painful. Mr. Green to day decided on exhibiting mercury, now that the man's health is in general respects improved, and as it was obvious the ulcers in different parts would not heal, nor probably assume any other appearance, unless the remedy were employed. He ordered unguent. hydr. ʒss. to be carefully rubbed in every night, and that the patient should not be distressed by the exercise required. Cat. lini, et Lotio chlorid. calcis, to the thigh.

14th. Sores on the legs have increased by sloughing, which is now at an end. Pulse is in some measure fuller, at 128, but not hard—skin warmer—no nightly sweats—bowels regular.

21st. The ulcer of the nose has spread on both sides, but its edges are clean and have tolerably red granulations. Very little alteration is observable in the general condition of the patient, which has hitherto varied much from day to day — the pulse, in particular, has been far different at one time from what it is at another; it is now beating 144. The mercurial friction and fumigation are persevered in, and the exertions disturb in a very trifling degree.

24th. Ulcer increasing on the face; those upon the extremities look well, and are diminished. His appetite is good, and he takes the nourishment allowed—sleeps well—pulse 140, of the same character.

28th. Diarrhœa again supervened yesterday but has abated. His strength, notwithstanding, is certainly augmented; the pulse, too, has become stronger; and the sores shew a much healthier surface, though that on the face has increased, and is increasing, and possesses an irritable and red circumference very favourable for the progress of ulceration.

31st. Has rested longer the last two nights than at any period since his admission. Sores are of almost the same appearance, and afford an abundant purulent secretion. The gums are sore and spongy; the rubbing in was intermitted last night by the sister in consequence, but Mr. Green has ordered it to be resumed. Wrists, and elbow of left side, have recently become enlarged, tender, and puffy, without external redness. Bowels quiet.

1831. Jan. 3d. A fresh ulcer has broken out on the right leg, sloughy and painful; the one occupying the face looks nearly as heretofore, being in some parts cleaner than in others. His appetite is excellent, and the pulse of more power. Bowels free. A strip of adhesive plaister has been applied over the ulceration and remaining half of the nose, to give the latter support; but as it produced too much irritation, it is discontinued. Poultices of linseed meal, and the chloride of lime lotion, have been constantly used to all the sores.

8th. The patient's appetite began to fail yesterday, and he finds himself weaker. The facial ulcer has extended in every direction by sloughing, and encroaches close upon the right eye; its surface is very foul; and the ossa nasi, the malar, and superior maxillary bones are exposed and dead. The margin is redder, and will probably soon be absorbed. Pulse is very feeble—the patient, however, is quiet, and does not endure much pain. Sores on the legs are healing fast, excepting the one which appeared last. Friction and fumigation are at present necessarily omitted under these circumstances, and Mr. Green remarked that so soon as the system was brought under the influence of mercury the diseases seemed to give way, in the improved state of the ulcers, but the constitutional powers proved insufficient to maintain the action of that remedy. He would now, therefore, endeavour to husband the strength, and perhaps he might again have the opportunity of giving the specific in question. Ordered. Extr. sarsæ, ʒj. Infus. calumb., ℥iss. Tinct. calumbæ, ʒj. M. t. d. Nutritious diet to be continued. Chloride of soda in solution, and poultices to the sore.

9th. Is better to day; has more appetite, and the wounds are looking cleaner; pulse has more power—bowels are very comfortably open.

11th. Feels more strength—pulse 130, fuller—appetite increasing. The sore is extending upon the cheek and base of the orbit.

18th. Some slight amendment has taken place in the patient's general health, but the ulcerative process is now advancing on both sides of the nose, and has come close to the left eye.

21st. Ulceration has extended, but the surface is rather improved and exhibits some granulations. Both ossa nasi are denuded even to their connexion with the frontal bone, and the skin immediately contiguous to the globe of the eye is ulcerated on the left side. Mr. Wall has seen the case, in order to try the effects of his remedy, but has no hopes of its being useful. He said it would be a needless waste of the medicine, but directed it to be employed in the following way. Poultices of bread and milk, in which a decoction of his vegetable is to be mixed up, twice a day; and the sore to be abluted with the decoction, when the cataplasms are removed.

31st. The appearance of the ulcer has become greatly improved, it being now fluid and granulating in one part, and its progress is arrested. In his general condition, the patient also finds himself better, and the ulcers upon the extremities are healing rapidly. Half an ounce of Mr. Wall's decoction is taken internally by his desire, and the other remedies are now discontinued by order of Mr. Green.

Feb. 8th. The patient is so much better that he has sat up for some hours together. He has taken abundance of nutriment, and the sore has put on a surprisingly healthy character. Mr. Wall has ordered three glasses of port wine daily, with two grains of sulphate of quinine in each, and an unlimited supply of fruit, &c.; but these were not sent from the apothecary's shop, being considered unnecessary.

14th. A relapse has occurred in the general health of the patient; and the ulcer is spreading on the right side of the face. Mr. Wall's treatment has been pursued entirely under his own superintendance.

18th. Some sloughing of the surface is further discernible upon the right side, but the nasal bones are covered with granulations. He complains of loss of strength, and rest; pulse 120, feeble; tongue clean; bowels open. The decoction is continued internally, and the wine; with the poultices and the lotion.

26th. No increase of sloughing can be seen. Patient thinks he has of late gained strength and appetite. Pulse 120, regular, and moderately full; tongue red at the apex, but clean; bowels not purged. Seeing the late return of sloughing, Mr. Wall has, within the last few days, abandoned the case. Lotio nigra and poultices of linseed meal have been again resorted to.

March 1st. The ulcer has not enlarged. He rests well, and has not lost strength. Mr. Green now prescribed Liq. hydr. oxymur. ♏xxx. Extr. sarsæ, ℨj. Decoct. sarsæ comp. ℥vj. bis quotidie.

Opium to be continued at night. Mutton chops, &c. have not been intermitted.

8th. In the vicinity of the right eye ulceration has in some degree spread, and the surface is foul. He perceives himself amended in the general state of health, and appetite is somewhat greater. Lotio hydr. flava, with the poultices.

22d. Patient has been much in the same way, and the ulceration is rather more favourable towards the eye, and in some parts there are seen small superficial sloughs, but the margin is clean. No healthy, or prominent granulations have sprung up. The tarsal border of the lower lid is implicated in the ulcerative surface, and we may say the external portion of the palpebræ is sacrificed. The medicines are still exhibited, hitherto without producing any sensible specific effect, and the bowels are in a tranquil state.

April 1. Progress of the ulceration has been stopped, and cicatrization has begun at the lowermost part; a spot of ulceration, not much larger than a shilling, remains just below the inferior lid. Same remedies continued.

9th. Patient has progressively improved in every respect, and now sits up. A considerable portion of the superior maxilla and turbinated bones has lately exfoliated, and a large vacuity is formed, leading from the face to the pharynx. An ulcer of healthy aspect and inconsiderable size exists on the right side of the face, a short distance below the tarsus.

30th. Great increase of flesh is visible; the ulceration is much contracted, and no further exfoliation has taken place. Redness of the gums is excited by mercury, and the oxymuriate is left off.

May 12th. Ulceration of the face is now cicatrized, but the deformity is of course very great. The entire nose is wanting, and an opening, larger than usual where this occurs, is presented to view. The sense of taste is perfect, and the hard palate is uninjured.

August 1st. He continues in the hospital, and has very much recruited in strength and plumpness. The integument adjoining to the aperture in the face remains sound, and likely to admit of the adaptation of a new nose from the forehead, which Mr. Green has in contemplation.

We were constant observers of this case, which was for the greater time indeed under our immediate care, and we cannot, in fairness, attribute the marked improvement which succeeded to the application of Mr. Wall to any specific influence upon the ulcer. The patient's system had just recovered from the debilitating effects of mercury, which were greater than it could withstand, by the employment of sarsaparilla and generous diet; and the condition of the surface was improving, together with the restoration of general constitutional power. Any simple and unirritating fluid, with poultices, at the same time, would have been attended with the like result. The little confidence in the efficacy of the remedy, shewn even by Mr. Wall, must be obvious in the report.

Case 3. *Excavated Bubo and Sinuses, depending on Constitutional Derangement. Trial of various Applications with only partial benefit.*

Dec. 31st, 1830. James Hearn, æt. 26, has been in the hospital many weeks, indeed since July 29th, for venereal complaints, which have been got rid of by an ordinary course of rubbing in and the local applications here used. Mercury has been discontinued since September. He has a large, irregularly shaped, and hollow ulcer in the left groin, originating in a bubo which was opened shortly after his admission. The edges of the ulcer are elevated much above the surface and extremely callous, and have been twice pared with the knife. It is rather narrow, passing obliquely down the fold of the groin, and then taking an horizontal course inwards towards the symphysis pubis, displaying very few and small granulations at the bottom, but there is a copious discharge of healthy pus from it. Endeavours to heal the sore have been made for some time with the red precipitate sprinkled upon the surface and dry lint then applied, a considerable degree of pressure being exerted at the same time against the hard and prominent margins by strapping. These means have been employed nearly a month, and we can observe only very slight amelioration, if, indeed, any, in the condition and aspect of the ulcerated parts. The patient has experienced pretty severe pain from it, particularly after each dressing.

The ulcer was to-day filled with wax, heated to the temperature, and in the manner recommended by Mr. Stafford; some strips of empl. resinæ were laid over the wax when cooled.

We were told by the patient in the evening that the application had afforded him greater ease than he had derived from any previous one, and the relief was uninterrupted.

1831, Jan. 2d. The wax-application has been attended with local ease till this morning, when we found the dressings loose, and the discharge, which was very abundant, mixed with the wax in a half-fluid state. These were removed, and the wound cleaned, but we could not yet perceive any improvement in it. Wax was re-applied, with the plaister, in the former mode.

6th. Says he has suffered more pain latterly from the present local remedy than he did from any other. A very profuse purulent discharge is daily taking place, and there is no change observable in the condition of the ulcer.

8th. Wax was again applied, after being kept on four days. The surface of the sore is certainly improved in appearance, but not contracted in dimensions.

18th. The edges are somewhat lower; the excavation appears less deep, and more inclined to produce healthy granulations. Continue.

22d. By Mr. Green's permission Mr. Wall visited the patient, and directed a decoction of his vegetable preparation to be mixed up with poultices of bread and milk, and these to be applied twice a day; after their removal, the surface to be also washed with the same liquid.

27th. The part has been comparatively free from pain; some whiteness is seen around the edges of the sore, attributed to the fluid used. Its surface, too, is cleaned by the poultices.

Feb. 1. The sides are still lower, and there is less excavation than we have yet witnessed, and the ulcer exhibits a more favourable aspect for healing.

8th. Sore has cicatrized in the centre, and the borders are more depressed. Pulvis cinchonæ, with the decoction and oatmeal poultices, has been employed to fill up the cavity under the direction of Mr. Wall.

16th. A large portion of new skin has been formed in the centre, connected by a narrow process to the surrounding integument; the elevated margins, formerly existing, are very materially altered both in height and their indurated character. Continue.

26th. Complains severely of pain in the ulcer. His countenance has become peculiarly pallid and wasted; general emaciation is likewise discernible; some cough, and other symptoms of pulmonic disorder.

March 11th. Constant pain is present in the ulcer, the condition of which is unaltered. Mr. Wall has not seen the man for many days, and consequently the dresser has had recourse to black-wash and poultices.

15th. The patient is forsaken by Mr. Wall, who has left the ulcer both larger and in a more foul condition than he found it. Equal parts of pulvis calaminæ and prepared chalk, with which the hollow sore is to be filled, have been ordered.

22d. Some inconvenience has arisen from the fetor produced by the powder absorbing the discharge, which, when dried, forms into cakes. The sore has been easier, but the surface has not been yet exposed to view since it was first covered over with the present remedy. Patient has some cough, and his countenance indicates organic disease within the chest, the features being contracted and sharp from wasting of flesh, and the eyes bright. But there is neither thoracic pain, nor dyspnœa, nor expectoration. His rest is broken at night; no nocturnal perspirations. Pulse 96, small, and weak. Tongue moist and clean. Bowels are rather obstinate, requiring house-medicine occasionally. Mr. Green prescribed Quin. sulph. gr. ij. Acid. sulph. dil. ♏ v. Infus. rosæ co. ℥j. M. ter quotidiè sumend.

April 6th. No improvement is manifest in the condition of the ulceration, but there has been some diminution of local pain. State of general health the same. The powder is omitted, and a lotion composed of oxide of zinc and lime-water (about ʒj. to the pint) is applied on lint three or four times daily. This is an application which Mr. Green has recently introduced, and employed beneficially in two or three instances of impetiginous affection of the skin. It seems to act as a local sedative, and purifies the parts with which it comes in contact.

28th. The ulcer is so far reduced in its dimensions that a line of granulations only exists at present, not more than an inch wide, of a florid colour, and clean. The lotion last specified has been continued, and patient's health would appear to be in a slight degree improved.

May 26th. Two or three sinuses have been opened, which led from the groin to the upper part of the scrotum; the old sores have advanced but little further in the process of cicatrization. His countenance remains of the same pallid hue. Lotion still employed, with poultices to the recent sores.

June 21st. The parts have been gradually healing with the same applications; three or four small and healthy ulcers are now observable in the midst of a large and irregularly-shaped cicatrix occupying the whole inguinal region.

July 12th. Fresh and sinuous ulceration has again taken place between the thighs and scrotum, from which there is a copious discharge of pus. The former sores are nearly well. Patient continues remarkably pallid; his looks are very unhealthy, but he has neither cough at present, nor pain of chest; the pulse is at 90, and small, and weak. The lotion is applied to the original sores, and linseed poultices to those which have lately broken out.

August 18th. The ulcers have somewhat contracted, and grown cleaner. But the health of the patient seeming to decline, he was now removed into the country by his own desire, and with the concurrence of Mr. Green.

The method of treating excavated ulcers with wax, as advised by Mr. Stafford, has been practised to some extent in this hospital, especially by Mr. Travers, and generally with good effect.

### II. Carcinomatous Ulceration of Mamma.

As we have commenced our present quarterly report with a remarkable instance of cancer, the following case may be here appended, demonstrating a fact which very rarely occurs, viz. the healing of a genuine carcinomatous ulcer.

Sarah Creed, 33 years of age, unmarried, and in a more respectable class of society than the greater number of hospital patients, applied at St. Thomas's Hospital, May 3d, 1831, and gave the subjoined account of a cancerous ulcer situated on the left breast.

As far back as seven years ago she observed a tumour on the right side of the nipple, the formation of which she is now inclined to assign to having taken cold after a wet journey; it was rather hard, and distinct upon manipulation from the natural structure of the organ; however, it acquired no magnitude and did not give rise to suffering, except an occasional lancinating pain at long intervals, and six months since it was not equal in size to a walnut. Subsequently to the latter distance of time she has consulted an irregular practitioner, and rubbed over the part an ointment that would appear to have been of a stimulant and irritating quality, as very soon after it was used ulceration of the integuments took place; and to the application she refers the early occurrence of this process. For the last three months, therefore, there has been a superficial ulcer on the left side of the nipple, which is now covered by a thin and vascular cicatrix of a red colour, a little larger than a half-crown-piece, and is surrounded by very great hardness and thickening of textures. Still nearer to the nipple, and on the same side, the skin is destroyed by a small slough, and on the right side a second exists, the nipple itself being entire, but in some degree sunken, or depressed, between them. The pain that has been experienced latterly cannot be considered severe, having principally occurred during the advancement of the sloughing action; and accordingly it has not been constant. The sound portions near to these sloughs are also ulcerated. Gravesend has been the residence of the patient. She has the appearance of a healthy woman; the breasts are large and well developed, and the catamenia have been perfectly regular; her spirits are low; she seems very anxious about her case; tears readily come into her eyes, and she often heaves a deep sigh whilst expressing regret at having lost so much time in the trial of useless and ill-advised remedies. Her appetite has been at no period as great as that of most people, but it has not recently declined; the tongue is moist and rather white, but not loaded; pulse 90, rather sharp—no thirst—bowels are in a free condition. Rest at night greatly broken, chiefly from mental excitement. Cat. panis bis die to the breast.

May 6th. Some little increase of sloughing is observable on the right side of the mamma, where more pain has been for a short time felt, but that on the left is separating and the surface underneath would seem to be granulating. A few hours rest was procured last night, but during the preceding two her eyes were scarcely closed. Continue.

23d. The slough is detached from the left side of the nipple, and the granulating surface has rapidly and perfectly cicatrized. Upon the right of the same process a large hollow space is formed by sloughing ulceration, and the excavation is tolerably clean, of a pale red colour. The surrounding hardness is somewhat less perceptible, and there has been very marked remission of pain, but three or four small glands are to be distinguished in a chain, commencing from below the nipple and passing towards the arm-pit; they are quite superficial, indurated, and destitute of pain or tenderness, though none reach into the axillary cavity itself. It has not been mentioned that we could discern no glandular swelling in this situation at her admission, and that the patient has never been sensible of any. Her general feelings have improved, together with a better appetite, and a more cheerful countenance. The bowels are in a regular state, and have not required the use of medicine. She has been taking, since the 10th, Pil. sapon. c. Opio, gr. v. o. n. which has procured ease and rest; and Extr. sarsæ, ℈j. Decoct. sarsæ comp. ℥vj. b. d. The present ulcer is covered twice daily with dry lapis calaminaris.

June 1. She declares herself quite free from pain, even at intervals. There is no manifest change either local or general, and the same remedial means are continued.

11th. The ulcer is contracted in size, and yields very little discharge. Some pain has been of late endured, and one of the axillary glands is swollen to the capacity of a filbert, but not tender nor painful. Pergat.

21st. No discharge has recently taken place from the ulcer, which is protected by a firm and excavated incrustation. To-day she has suffered from severe pain situated a little above the nipple, the part being also heated. The state of the glands is not altered. A lotion of spirit of wine was ordered to be applied to the seat of pain.

28th. The pain has ceased, and the ulceration, although truly cancerous, is quite healed, the cicatrix being thin, and perhaps more properly a crust. But a more distinct and deeply-seated hardness is felt within the substance of the breast, branching out in various directions from the nipple, more especially upwards. The superficial chain of glands below have a reddened surface, and are very tender. Patient's general health is still unaffected; in fact, she avers it is much better, and her spirits are excellent, she believing (would we could do the same!) that her breast will soon be cured. Same local applications.

July 1st. The pain has again increased. Twelve leeches to be applied.

6th. Has found complete relief from the leeches; some pain remains in another part of the gland, with slight local heat. Cold lotions continued, with the pulv. calaminæ.

12th. Owing to augmentation of pain, the leeches have been repeated, and procured effectual alleviation. Greater superficial redness of the smaller enlargements has been latterly observable, which last are encroaching towards the right mamma. Her internal medicines and the topical remedies are persevered with.

21st. She was made an extra-patient this day, partly at her own request. An ichorous discharge has been flowing from the circumference of the mammilla, where there is a slight fissure. Her mixture and pill are to be continued; and the pulv. calaminæ applied to the nipple. She returned to Gravesend.

August 19th. The patient appeared at the hospital to day; little or no change is manifest in the state of the disease, and her health is corrected by residence in the country. Same means were directed by Mr. Green to be continued.

That the disease will ultimately prove mortal, no one who has witnessed it can doubt. At the same time, the temporary, though complete, cicatrization of the sore, has tended in no small measure to allay the poor woman's distress and anxiety of mind; and thus her life has been prolonged, and her sufferings, far from being aggrandized, are now not worthy of mention.—Beta.

III. Aneurism of the Aorta bursting through the Left Lung into the Thorax.

Edmund Nicholson, a fine muscular man, was admitted into St. Thomas's Hospital, April 7th, 1831. He stated that, on the 28th and 31st. of the preceeding month, he had brought up a large quantity of blood during a violent fit of coughing. According to his account he brought up ℔j. on the 28th, and as much as ℔iij. of blood on the 31st.—the first blood coughed up was arterial—on the second occasion it was dark and coagulated. He has now cough, which is occasional, slight, and teazing, but does not come on in violent paroxysms—small quantities of coagulated blood are frequently brought up. By the stethoscope there is found to be too little respiration over the front and upper half of the left side of the chest, with a crepitous rattle. He says he has occasional palpitation of the heart—and that he has been ill a year.

He was immediately cupped to ℥xij. —and, during this and the following month, blood was extracted, either by the cupping-glasses or lancet, every 3d or 4th day; sometimes even more frequently. A succession of blisters were applied to the chest, and he was kept on very spare diet. By this rigorous plan of treatment he so far recovered as to leave the hospital on the 23d of June, but on the 7th of July he again presented himself, having been suddenly seized with a fit of coughing, during which he brought up a large quantity of blood: he was immediately bled to ℥xvj. and had cold fluids given to drink. About 9 o'clock he had further hæmorrhage—was ordered plumbi acet. gr. j., opii, gr. $\frac{1}{4}$, 4tis horis, and to have thirty leeches applied to the chest. He became tranquil, and had some sleep during the night—but early on the morning of the 8th he had another violent fit of coughing, brought up above a quart of arterial blood, and immediately expired.

*Sectio Cadaveris, seven hours after death.* The body was completely blanched. On removing the sternum and cartilages of the ribs, the left pleural sac was found completely filled with partially coagulated arterial blood. The upper part of the lung felt dense, elastic, of a very unusual globular form, and much increased in volume. When the blood had been removed (of which there was between 2 and 3℔.) a rent was found in the anterior part of the upper lobe of the lung, of sufficient size to admit a finger to pass with ease to a large cavity, into which the upper part of this viscus was converted—and from which the fatal hæmorrhage had taken place. The edges of the opening were ragged, and quite recent. Some old adhesions were divided, and the whole contents of the thorax then removed. The aorta was next slit up, when a smooth circular opening of an inch diameter was found in the concavity of the arch, just as it turns round the root of the left lung, from which a finger could be passed into the large cavity just described, and consequently into the thorax, by the rent of the lung. It was now apparent, that the globular appearance of the upper half of the lung had for its cause an aneurism of the aorta imbedded in its substance; the direction and progress being determined by the proximity of the root of the lung, and especially of the origin of the left bronchial tube. The aneurismal sac and pleura pulmonalis alone remained at the most superior part; the pulmonary structure having entirely disappeared by progressive absorption: this part was also the thickest of the cavity. The rent was at the lowest part of the cavity, and in this situation the sac was thinner than elsewhere. A very close adhesion had taken place between the origin of the aneurismal sac and the bronchial tube, and ulceration of one cartilaginous ring, for a line and half in extent, had taken place. A small quantity of blood was found in the left bronchial tubes, as well as in the parenchyma of the lung. The heart was not larger than might be expected in so muscular a man; and the arterial system was remarkably free from disease.

IV. PARAPLEGIA FROM SCROFULOUS TUBERCLE IN SPINAL CHORD.

Daniel Rennet, 9 years of age, of scrofulous appearance, was admitted into St. Thomas's Hospital, April 15th, 1831, for paralysis of the inferior extremities. It was stated that the paralysis had come on rather suddenly, and that it had affected the left leg four weeks ago; the right retaining its power until one week ago. He has now entirely lost all power of motion and sensation of the extremities and trunk, as high as the sides of the thorax—has no pain of the back—there is no irregularity of the spine—nor does he complain of any pain on pressing or striking the vertebræ. The limbs are contracted towards the trunk, and are rigid. He complains of constant pain of the abdomen—bowels constipated—appetite rather voracious. He had simple aperient medicine given in the first place, and was then put under the influence of mercury, but without any benefit being derived, the strychnine was then commenced in doses of one-twelfth of a grain three times a day, and steadily increased in quantity until he took the one-sixth of a grain in each dose. The full effect of the medicine was produced, viz:—frequent severe spasms of the muscles of the lower extremities, by which they were forcibly jerked up to the trunk, accompanied with considerable pain: the dose was diminished, and persisted in during a month, but without any amendment. The paralysis is undiminished—his abdomen became swollen and tumid—emaciation took place, notwithstanding wine and generous diet. Sloughing of the nates took place, and he died June 22d. In the post-mortem examination, a scrofulous tubercle was found imbedded in the spinal chord, in the situation of the second dorsal vertebra. Its situation was in the substance of the chord itself, the membranes being scarcely adherent, and turned down with the greatest facility; the *whole* of the medullary matter originally occupying the situation of the tubercle had been absorbed, leaving it (the tubercle) and the membranes, the only media of connexion betwixt the upper and lower portions of the chord. The size of the tubercle was that of a large hazle-nut; the colour yellow; structure firm, with the centre a little softened; presenting all the characters of an ordinary scrofulous tumour. The chord below the situation of the deposit was of usual size and texture. The mesenteric glands were all much enlarged, and contained sundry scrofulous matter in their interior—some had gone on to the formation of pus. There was likewise some scrofulous matter deposited in the lower part of the right lung.—Delta.

